# Dual-band mixed-mode bandpass filter based on dual-layer substrate integrated waveguide resonator loaded with A capacitive patch

**DOI:** 10.1371/journal.pone.0330712

**Published:** 2025-09-08

**Authors:** Rihui Zeng, Jianpei Chen, Xia Luo, Dongya Shen, Chuanxi Xing

**Affiliations:** 1 The School of Electrics and Information Engineering, Yunnan Minzu University, Kunming, China; 2 The Yunnan Key Laboratory of Unmanned Autonomous System, Kunming, China; 3 Yunnan Provincial Engineering Laboratory of Cloud Wireless Access & Heterogeneous Networks, Yunnan University, Kunming, China; Zhejiang University, CHINA

## Abstract

To address the growing demand for compact and high-performance microwave filters in modern communication systems, a mixed-mode bandpass filter is proposed in the article. A dual-layer substrate integrated waveguide resonator loaded with a capacitive patch (CP-DSIWR) is proposed and theoretically analyzed, with both patch modes and cavity modes existing. To construct the bandpass filter, two rows of metallic vias are designed in the CP-SIWR to enable coupling between the two types of the modes, with the structure being fed by microstrip line. A slot is embedded in the center of the capacitive patch to tune the resonant frequencies. The simulated results of the filter show that TM_01_, TM_10_ and TE_101_ modes are excited, achieving a dual-band filtering response with tunable frequency characteristics. The filter is centered at 6.2 GHz and 12.9 GHz with a compact size of 0.57$\lambda_{g}$×0.57$\lambda_{g}$. A prototype is fabricated and measured for validation, showing good agreement between the simulation and measured results.

## 1 Introduction

With the rapid advancement of wireless communication systems, the demand for compact and high-performance multi-band filters is increasing. A technology of planar microwave circuits, substrate integrated waveguide (SIW), is characterized by its low cost, low transmission loss, compact size, and excellent compatibility with other planar microwave circuits, has become prevalent in the design of highly reliable planer filters [1–2]. Traditional SIW bandpass filters, designed based on the fundamental TE_101_ mode [3–4], posed challenges in achieving compact multi-band filtering characteristics.

To address these limitations, researchers have explored various methods to enable multi-mode and multi-band operation. Typically, there are three primary methods for implementing multi-band bandpass filters. The straightforward approach involves forming multiple coupling paths between the source and load by combining independent single-band filters, which has been extensively implemented on SIW and microstrip platforms [5–9]. However, the strategy for multi-band filter results in relatively larger circuit size. An alternative method entails dividing a wide passband into several sub-passbands by introducing multiple transmission zeros. This technique has been explored for achieving dual- and tri-band filters [10–13], although it is limited to specific frequency ratios.

To solve the problem, an advanced approach leveraging dual- or tri-mode resonators has emerged, offering advantages such as compact size and enhanced design flexibility [14–17]. A dual-band dual-mode bandpass SIW filter with wide bandwidth and controllable transmission zeros was presented, which used two modes without adjustable center frequency [14]. Another work introduced two dual-band filters within a single cavity through overlapping mode control, achieving excellent frequency ratios [15]. A dual-band quad-mode SIW filter with multi-mode characteristics was reported in [16], and a quad-mode quad-band filter was reported in [17]. Despite their high selectivity and excellent out-of-band rejection, these designs exhibit large insertion loss at lower frequency, such as 2.21 dB in [16] and 2.39 dB in [17].

In the paper, a dual-layer substrate integrated waveguide resonator loaded with a capacitive patch (CP-DSIWR) is proposed to introduce both patch modes and cavity modes, thereby significantly increasing the number of available resonant modes. Based on the technology of CP-DSIWR, a dual-band mixed-mode bandpass filter is constructed. The capacitive patch is designed to generate additional patch modes when fed appropriately, ensuring the simultaneous presence of both patch modes and cavity modes within the resonator. By embedding a slot on the capacitive patch, the frequencies of these resonant mixed-modes can be controlled, and a transmission zero is introduced to the right of second passband.

## 2 Mixed-mode bandpass filter theory and analysis

### 2.1 Analysis and design of dual-layer substrate integrated waveguide resonator loaded capacitive patch

Generally, the resonant modes existing in a SIW cavity are limited to cavity modes. In this section, an improved SIW cavity is constructed by introducing a capacitive patch between two vertically stacked SIW cavities, forming what is referred to as a dual-layer substrate integrated waveguide resonator loaded with a capacitive patch (CP-DSIWR). In the CP-DSIWR, both patch modes and cavity modes exist.

**Fig 1:**
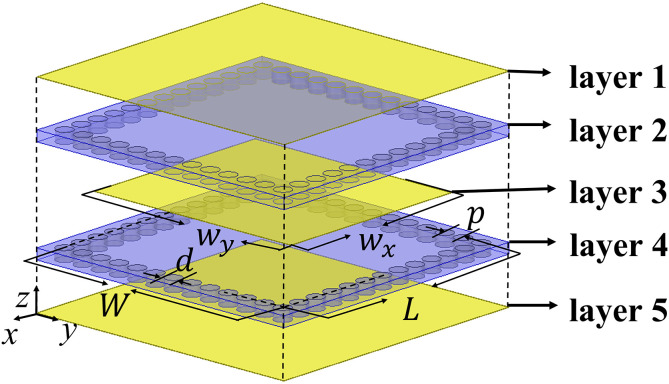
Dual-layer substrate integrated waveguide resonator loaded with a capacitive patch (yellow: copper; light blue: dielectric substrate; blue: metallic vias).

The configuration of the proposed CP-DSIWR, illustrated in Fig 1, consists of two dielectric layers and three conductive layers. The dielectric substrates, layer 2 and the layer 4, are Rogers RT5880, with a relative permittivity $\varepsilon_{r\;}=2.2$, a loss tangent tanδ=0.0009, dimensions of W×L and a thickness of 0.508 mm. Layer 1 and the layer 5 form the top and bottom conducting surfaces of the resonant cavity, respectively. Layer 3 is the loaded capacitive patch with dimensions of $w_{x}\times w_{y}$. For all examples presented in this paper, the diameter of the metallic vias is selected as d=0.8 mm, and the pitch between adjacent vias is set to p=1 mm.

To investigate the effects of the capacitive patch on resonant modes in the CP-DSIWR, a comparison is conducted between the proposed CP-DSIWR and a traditional SIW resonator with the same physical dimensions. Both structures are simulated and analyzed in the software ANSYS with the eigenmode solver.

**Fig 2:**
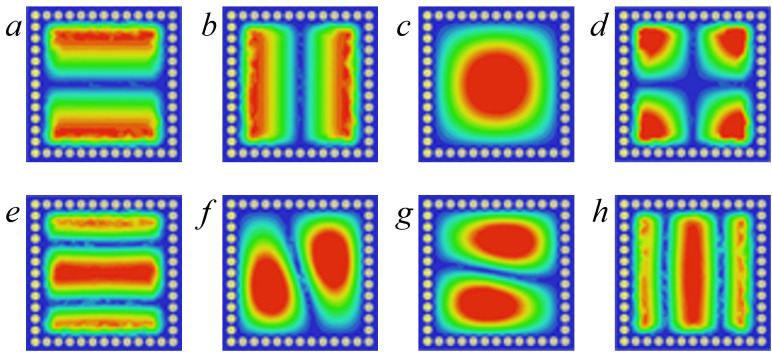
The electric field distributions of the modes for the proposed CP-DSIWR (Resonant frequency from low to high). (a)TM_01_; (b)TM_10_; (c)TE_101_; (d)TM_11_; (e)TM_**02**_; (f)TE_201_; (g)TE_102_; and (h)TM_20_.

To explore the frequency characteristics of the patch modes and cavity modes in the proposed CP-DSIWR, the electric field distributions of the resonant modes for the CP-DSIWR within frequency range of 0 ~ 20 GHz are simulated, as shown in Fig 2. The simulated electric field distributions reveal that eight eigenmodes exist in the CP-DSIWR: (a)TM_01_, (b)TM_10_, (c)TE_101_, (d)TM_11_, (e)TM_02_, (f)TE_201_, (g)TE_102_ and (h)TM_20_, which including both cavity modes corresponding to TE modes and patch modes corresponding to TM modes.

**Fig 3:**
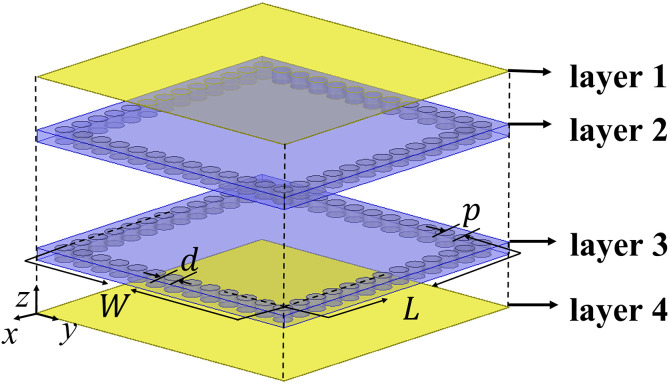
The configuration of traditional substrate integrated waveguide (yellow: copper; light blue: dielectric substrate; blue: metallic vias).

**Fig 4:**
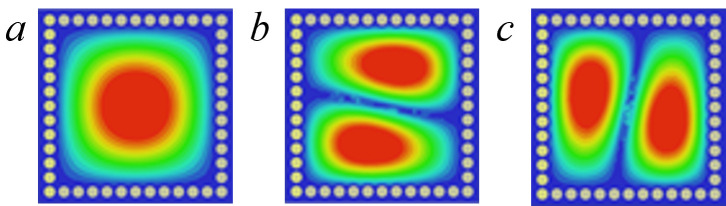
The electric field distributions of the modes for the traditional SIW resonator (Resonant frequency from low to high). (a) TE_**101**_; **(b) TE**_**102**_**; and (c) TE**_**201**_.

To highlight the advantages of the proposed CP-DSIWR, its performance is compared with that of a traditional SIW resonator. Also, within the frequency range of 0 ~ 20 GHz, and with identical physical dimensions of W×L and a thickness of 1.016 mm, the traditional SIW resonator is modeled and simulated. The electric field distributions of the resonant modes for the traditional SIW resonator are shown in Fig 3, and only three TE modes (TE_101_, TE_102_ and TE201) exist which are shown in Fig 4. Compared with the traditional SIW resonator, there are five additional resonant modes in the CP-DSIWR, which demonstrating its mixed-mode resonant characteristics. The loaded capacitive patch in the CP-DSIWR not only generates additional patch modes but also influences the eigenfrequencies of the resonator.

**Fig 5:**
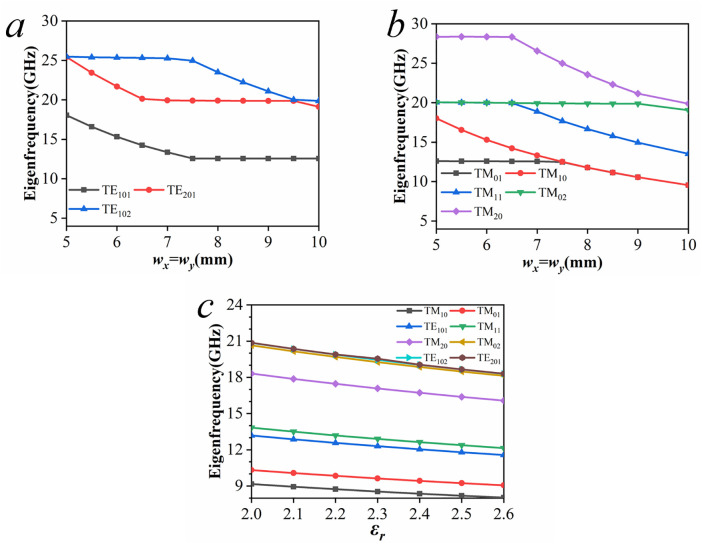
Eigenfrequencies. (a) The cavity modes with varying $w_{x}$ and $w_{y}$ when $w_{x}=w_{y}$; (b) The patch modes with varying $w_{x}$ and $w_{y}$ when $w_{x}=w_{y}$; and (c) The cavity modes and the patch modes with varying $\epsilon_{r}$.

To study the effects of dimension of the loaded capacitive patch and the relative permittivity of the dielectric substrates on resonant modes in the CP-DSIWR, the simulations of the resonator with different values of the patch dimensions $w_{x}$ and $w_{y}$ when $w_{x}=w_{y}$ and various $\varepsilon_{r}$ are performed. The simulated results of eight modes in Fig 2 are categorized into three cavity modes (TE_101_, TE_102_, TE_201_) in Fig 5(a) and five patch modes (TM_01_, TM_10_, TM_11_, TM_02_, TM_20_) in Fig 5(b). In these figures, the impact of the patch dimensions on the resonant modes is analyzed. Furthermore, Fig 5(c) illustrates the effect of the relative permittivity on these eight modes, providing insights into how variations in material property affect their resonant behavior.

As indicated in Fig 5(a), when $w_{x}$ and $w_{y}$ vary from 5 mm (0.23$\lambda_{g}$) to 10 mm (0.47$\lambda_{g}$), the eigenfrequencies of the cavity modes decrease. The guided wavelength $\lambda_{g}$ in the CP-DSIWR at the fundamental cavity mode frequency 12.8 GHz can be calculated by (1), where λ is the vacuum wavelength, $\varepsilon_{r}$ is the relative permittivity of the dielectric substrate, W and L are the distances between the centers of the outermost vias along the width and length directions, respectively, and $W_{eff}$ and $L_{eff}$ are the equivalent width and length of the proposed CP-DSIWR, can be calculated by (2) [18].


$$\lambda_{g}=\frac{\lambda }{\sqrt{\varepsilon_{r}-{\left(\frac{\lambda }{2W_{eff}}\right)}^{2}}}$$ (1)

$$W_{eff}=W-\frac{d^{2}}{0.95p},\;L_{eff}=L-\frac{d^{2}}{0.95p}$$ (2)

As shown in the simulated results of Fig 5(b), the eigenfrequencies of the patch modes also decrease with increasing sizes of the loaded capacitive patch, which can be explained with the stripline resonant theory. The resonant frequency $f_{TM10}$ of the TM_10_ mode can be expressed as [19].


$$f_{TM10}=\frac{1}{2w_{x}\sqrt{\varepsilon_{r}}\sqrt{\varepsilon_{0}\mu_{0}}}$$ (3)

where $\varepsilon_{0}$ and $\mu_{0}$ are the vacuum permittivity and vacuum permeability, respectively.

To utilize the TE_101_ mode to design a bandpass filter, its eigenfrequency characteristic are studied in detail and significant conclusion can be found from Fig 5(a). As the patch size $w_{x}\times w_{y}$ reach up to 7.5 mm × 7.5 mm (0.35$\lambda_{g}$×0.35$\lambda_{g}$), the eigenfrequency of the TE_101_ mode decreases linearly, due to the change of electric field distribution for the cavity mode TE_101_ by the increasing size of the capacitive patch. However, once the capacitive patch size exceeds 0.35$\lambda_{g}$×0.35$\lambda_{g}$, the eigenfrequency of the TE_101_ mode remains almost constant, same with the case of the traditional SIW resonator. This behavior can be attributed to capacitive loading saturation, which occurs when the capacitive loading effect reaches a limit, while additional coverage of the capacitive patch almost does not introduce changes of electromagnetic field distribution. In the state of capacitive loading saturation, further increases in patch size have minimal effect. Therefore, the field distribution of the TE_101_ mode becomes predominantly determined by the traditional SIW structure. Consequently, the eigenfrequency characteristics of the TE_101_ mode in the CP-DSIWR stabilizes and behaves similar to those of the TE_101_ mode in a traditional SIW resonator.

Based on the above analysis, the resonant frequency of the TE_101_ mode can be revised based on that of the traditional SIW resonator. When the capacitive patch size (CPS) is smaller than 0.35$\lambda_{g}$ ×0.35$\lambda_{g}$, its eigenfrequency can be approximated in (4), which was derived through multiple fitting calculations. Conversely, when the capacitive patch size exceeds 0.35$\lambda_{g}$×0.35$\lambda_{g}$, the eigenfrequency of the TE_101_ mode is approximated in (4) [1].


$$f_{TE101}=\left\{ \begin{array}{c} \frac{7W_{eff}}{12w_{x}}\frac{c}{2\sqrt{{\varepsilon_{r}\mu }_{r}}}\sqrt{{\left(\frac{1}{W_{eff}}\right)}^{2}+{\left(\frac{1}{L_{eff}}\right)}^{2}}\;{{CPS<(0.35\lambda }_{g})}^{2}\\ \frac{c}{2\sqrt{{\varepsilon_{r}\mu }_{r}}}\sqrt{{\left(\frac{1}{W_{eff}}\right)}^{2}+{\left(\frac{1}{L_{eff}}\right)}^{2}}\;CPS>{{(0.35\lambda }_{g})}^{2} \end{array}\right.\;$$ (4)

where c is the speed of light in vacuum, and $\mu_{r}$ is relative permeability of the dielectric substrate.

As observed from Fig 5(a), the eigenfrequency characteristics of the TE_102_ and TE_201_ modes exhibit a complementary downward trend that depends on the dimensions of the capacitive patch. The TE_102_ mode maintains a nearly constant frequency with increasing CPS until 7 mm × 7 mm (0.33$\lambda_{g}\times$0.33$\lambda_{g}$). Beyond the size, the eigenfrequency of TE_102_ decreases as the loaded capacitive patch perturbs the dual-peak electric field distribution for the TE_10_ mode. With a complementary downward trend, the TE_201_ mode presents frequency reduction with increasing patch size smaller than 7 mm × 7 mm, due to orthogonal field pattern with TE_102_. This delayed decreasing behavior of TE_102_ versus TE_201_ originate from their differences of electric field distribution. When the loaded capacitive patch sufficiently covers their respective mode electric field more than 85%, the eigenfrequencies characteristics of TE_102_ and TE_201_ maintain constant. Fig 5(c) illustrates that both patch modes and cavity modes exhibit decreasing eigenfrequencies with increasing relative permittivity$\varepsilon_{r}$, consistent with the theoretical predictions from (3) and (4). Notably, TM modes show smaller frequency shifts (TM_10_: 1.12 GHz) compared to TE modes (TE_101_: 1.62 GHz), and high-order modes such as TM_20_ (2.24 GHz) and TM_02_ (2.53 GHz) exhibit even larger frequency shifts, with $\varepsilon_{r}$ varying from 2.0 to 2.6. So, high-order mode indices require more stricter control over substrate permittivity to obtain stable frequency performance in multi-mode SIWR designs. The above analysis provides predictable physical parameters and material property of the CP-DSIWR to design multi-band filter.

For the capacitive patch size of $w_{x}=w_{y}=8$ mm and the relative permittivity of $\varepsilon_{r}=2.2$, the unload quality factors ($Q_{u}$) of the first three resonant modes with the resonant frequency around 15 GHz in the CP-DSIWR are simulated using HFSS, as shown in Table 1. Due to the nearly enclosed cavity structure, these modes possess relatively high $Q_{u}$.

**Table 1:** Unload quality factors of CP-DSIWR.

Mode	TM_01_	TM_10_	TE_101_
$Q_{u}$	454	455	572

### 2.2 Design of dual-band bandpass filter

#### 2.2.1 Mixed-mode bandpass filter.

**Fig 6:**
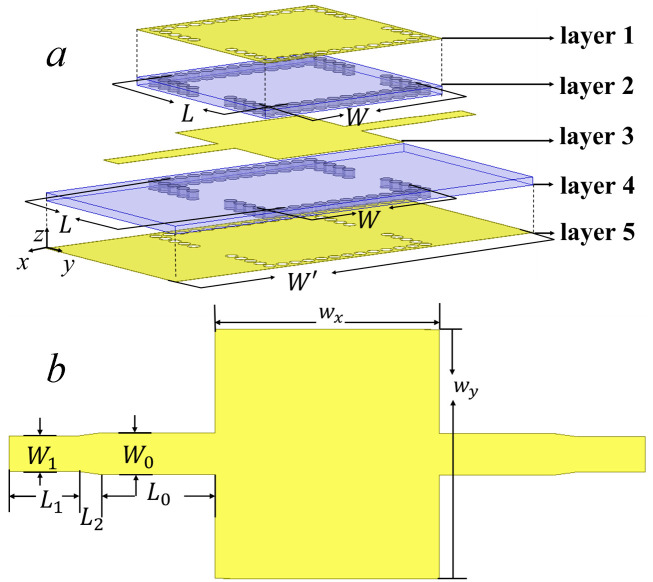
Direct feeding of the capacitive patch. (a) Filter configuration; and (b) The configuration of the layer 3.

**Fig 7:**
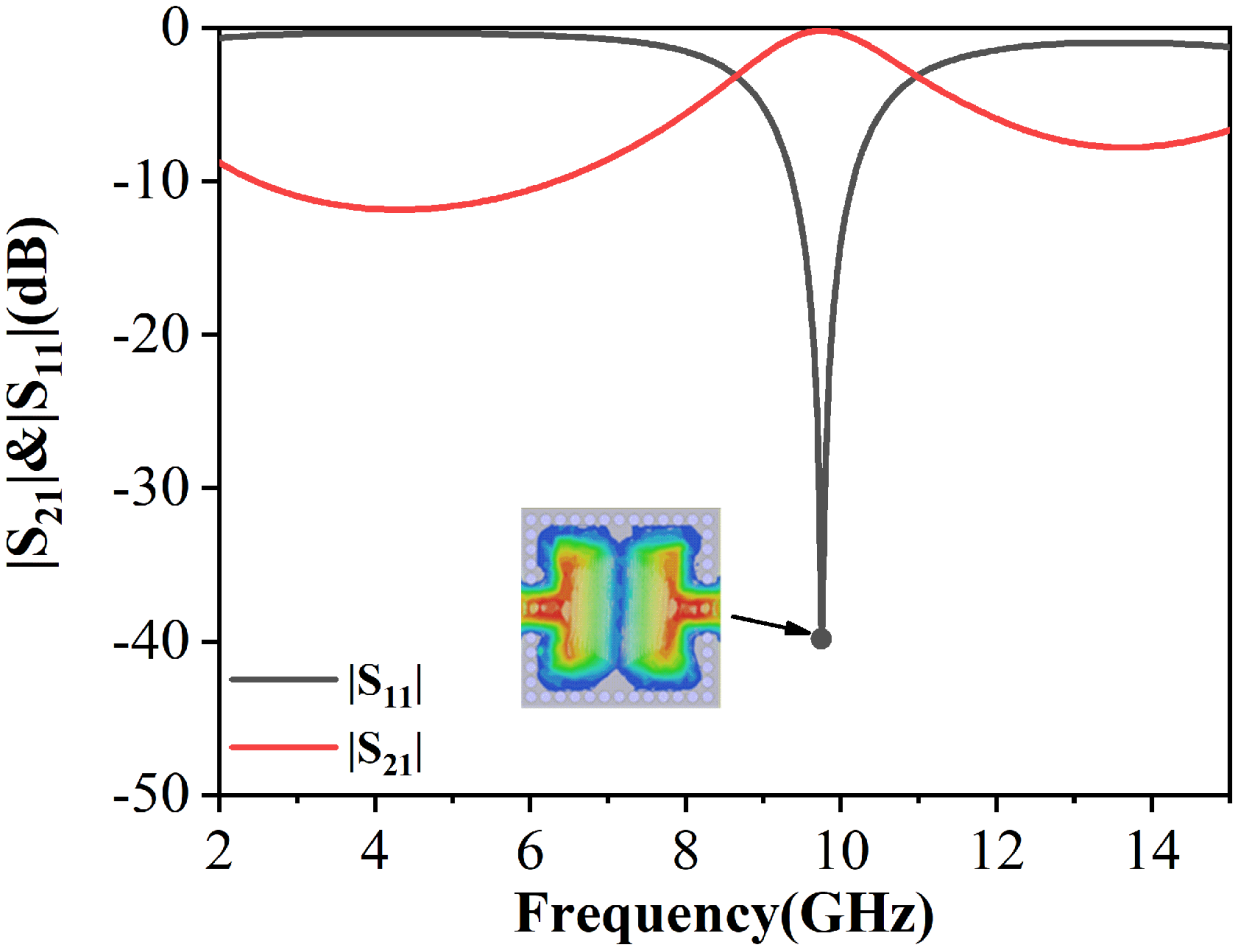
Simulated responses of direct feeding of the capacitive patch when $\begin{array}{c} \;w_{x}\;=\;9.7 \end{array}$ mm and $\begin{array}{c} {𝐰}_{y}\;=10.8 \end{array}$ mm.

According to the analysis in Section 2, a direct-fed CP-DSIWR filter is designed, as shown in Fig 6(a). The filter consists of a CP-DSIWR with a pair of input and output direct feeding structures connected to the capacitive patch. Each feeding structure consists of a 50 Ω microstrip line and a transition structure. Fig 6(b) show the structure of layer 3, which features a central patch connects to a 50 Ω microstrip feedline of width $W_{1}$ and length $L_{1}$through a tapered section of length $L_{2}$ and transitions to a stripline with width $W_{0}$ and length $L_{0}$. This integrated feeding design ensures good impedance matching from patch to feedline. Fig 7 shows the simulated S-parameters for this feeding configuration, demonstrating the excitation of the TM_10_ mode.

To excite both patch modes and cavity modes, the direct-fed CP-DSIWR filter is revised by incorporating two rows of periodic metallic vias with diameter $d_{1}$=0.8 mm and pitch $p_{1}$=1 mm positioned centrally in the layer 4. The coupling between the patch modes and cavity modes is achieved by electrically connecting the capacitive patch to the ground plane through these metallic vias. Specifically, the metallic vias serve as conductive paths that connect the capacitive patch to the ground plane, through which the electric fields of the patch modes and cavity modes is redistributed and propagated to the ground plane, forming new coupling paths. The propose structure enables the realization of a mixed-mode bandpass filter utilizing both the cavity and patch modes of the proposed CP-DSIWR.

**Fig 8:**
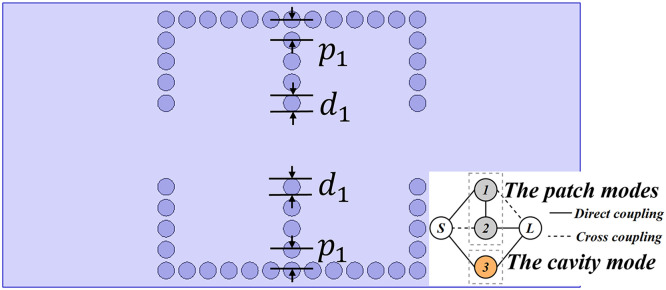
The configuration of layer 4 of the mixed-mode bandpass filter and the coupling topology.

Fig 8 shows the structure of the layer 4 of the proposed mixed-mode bandpass filter with its coupling topology. In the layer 4, two rows of metallic vias with diameter d are arranged in the center of the bottom cavity, which facilitates the coupling between the patch modes and the cavity modes, enabling effective mixed-mode operation. The coupling topology, shown in bottom of Fig 8, where nodes 1 and 2 represent the TM_01_ and TM_10_ patch modes, and nodes 3 represent the TE_101_ cavity mode. The input and output are defined by nodes S and L, respectively.

**Fig 9:**
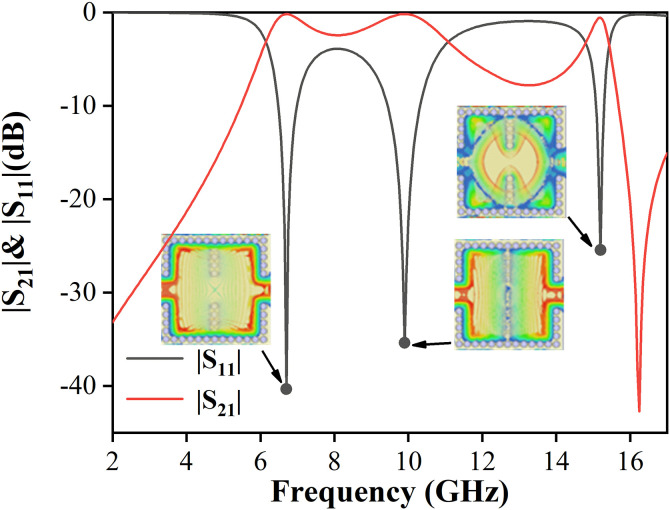
Simulated responses of the mixed-mode bandpass filter when $\;w_{x}\;=\;9.7$ mm and ${𝐰}_{y}\;=10.8$ mm.

Fig 9 presents the simulated S-parameters and the electric field distributions of the first three resonant modes. Compared to the configuration without metallic vias, the addition of two rows of metallic vias successfully excites additional modes: the TM_01_ patch mode and the TE_101_ cavity mode. This confirms that the proposed filter design with mixed-mode excitation is capable of simultaneously exciting both patch and cavity modes. The TM_01_, TM_10_ and TE_101_ modes corresponding to (a), (b), and (c) in Fig 2, are operating in the mixed-mode bandpass filter.

**Fig 10:**
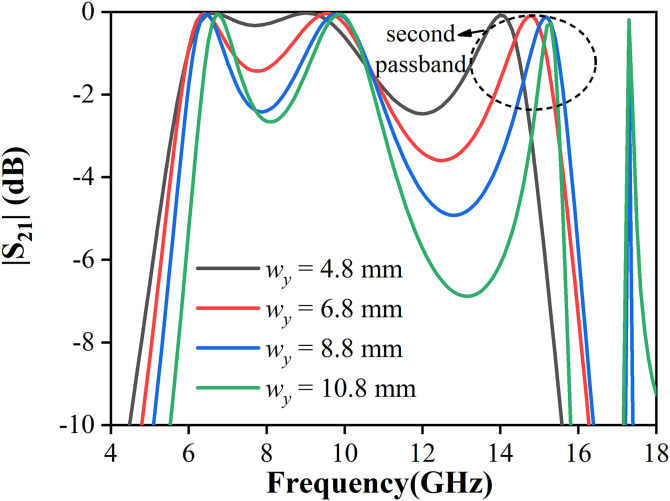
Simulated |S_21_| of passband against ${𝐰}_{y}$ with unchanged ${𝐰}_{x}\;=\;9.7$ mm.

Fig 10 shows the simulated S-parameter versus the length $w_{y}$ of the capacitive patch. It can be seen that the resonant frequency of TE_101_ increases as $w_{y}$ increases. However, the bandwidth of the second passband decreases, indicating that the size of the capacitive patch affects the cavity modes.

The final dimensions of the mixed-mode bandpass filter are as follows: W’=30 mm, L=12 mm, $W_{0}=1.8$ mm, $W_{1}=1.57$ mm, $L_{0}=4.95$ mm, $L_{1}=3$ mm, $L_{2}=2.2$ mm, $w_{y}=10.8$ mm, $w_{x}=9.7$ mm.

#### 2.2.2 Dual-band mixed-mode Bandpass Filter.

**Fig 11:**
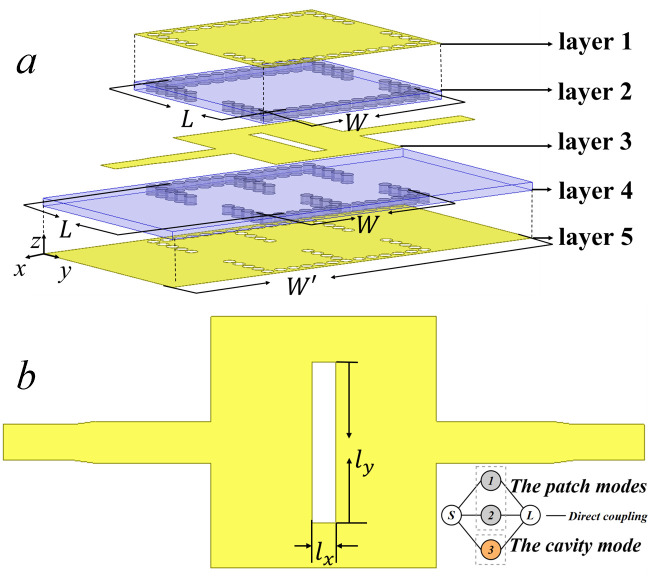
Dual-band mixed-mode bandpass filter. (a) Filter configuration; and (b) The configuration of layer 3 and the coupling topology.

Based on the mixed-mode filter in subsection 2.2.1, a dual-band mixed-mode bandpass filter is proposed by introducing a slot in the center of the layer 3 of the capacitive patch. As shown in Fig 11(a) and 11(b), the design features a slotted capacitive patch to strategically tune resonant frequencies by altering current distribution, enabling precise control over the coupling strength between the TM_01_ and TM_10_ patch modes and altering the coupling path to achieve dual-band performance.

**Fig 12:**
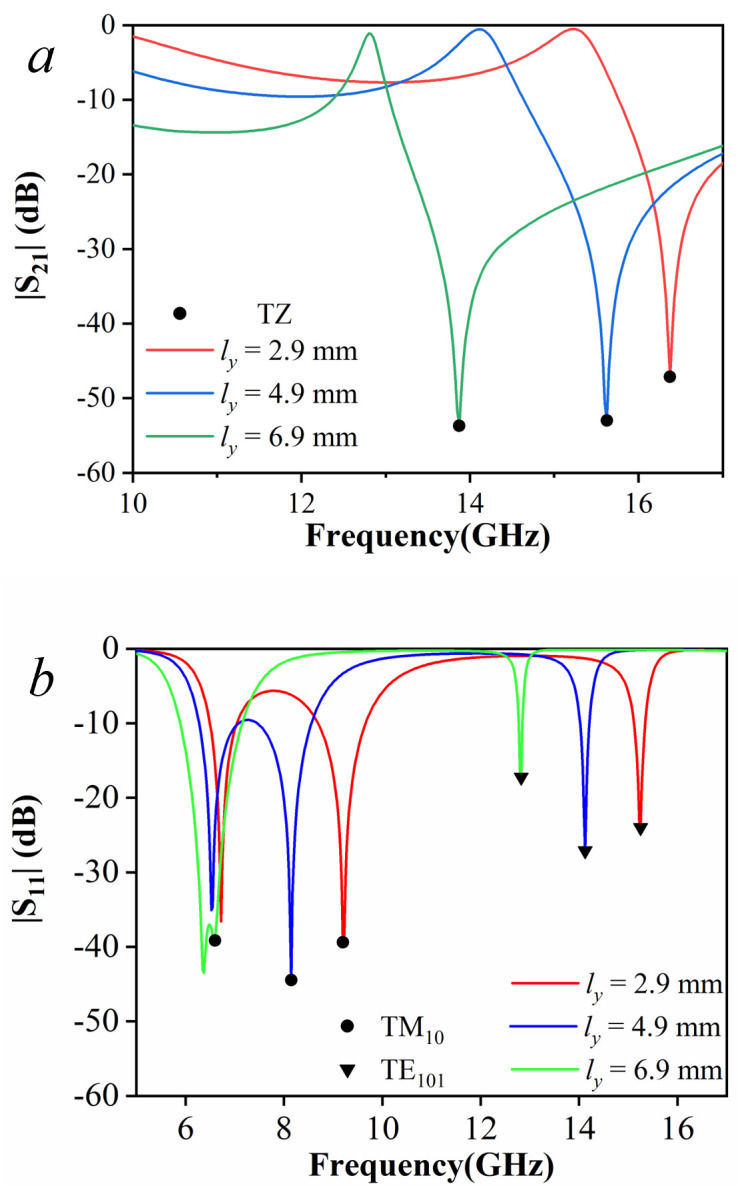
S-parameters. (a) |S_21_|; and (b) |S_11_|.

Fig 12 presents the variations of the S-parameters versus the parameter $l_{y}$. The slot controls both TM_10_ and TE_101_ resonant frequencies tuning and the transmission zero positioning. As shown in Fig 12(a), the transmission zero, marked by circular markers, shifts to lower frequency and stabilizes in the upper stopband of the second passband as $l_{y}$ increases. Meanwhile, Fig 12(b) demonstrates that with increasing $l_{y}$, the slot is introduced to optimize passband formation by shifting the resonant frequency of TM_10_ mode toward TM_01_ mode, marked by circular markers and triangle markers respectively, to form a passband.

**Fig 13:**
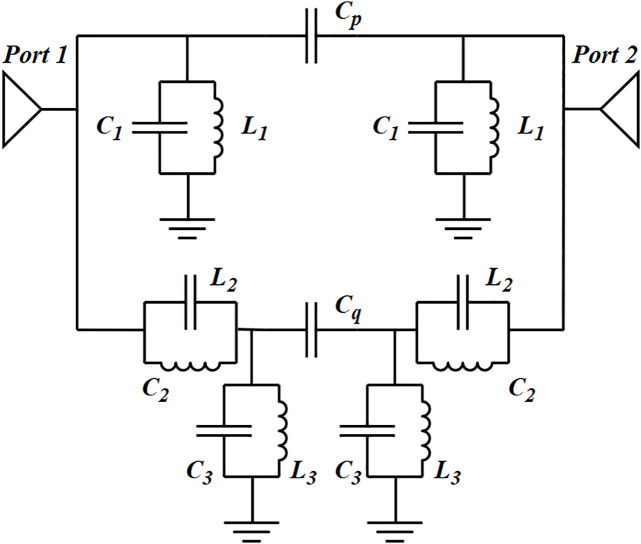
The equivalent-circuit model of the dual-band mixed-mode bandpass filter (${𝐂}_{p}=0.21$ pF,$\;C_{q}=0.29$ pF, ${𝐋}_{1}=0.5$ nH,$\;C_{1}=1.05$ pF, ${𝐋}_{2}=0.12$ nH, ${𝐂}_{2}=0.73$ pF, ${𝐋}_{3}=0.8$ nH, ${𝐂}_{3}=0.5$ pF).

To further analyze the operational principles of the dual-band mixed-mode bandpass filter, an equivalent-circuit model is designed which is shown in Fig 13. The first passband is generated by the effect of the slot on the coupling of TM_01_ and TM_10_, which can be equivalent as a resonant circuit represented by the capacitor $C_{3}$ and the inductor $L_{3}$. The second passband is generated by the resonance of the cavity mode TE_101_, which is equivalent to the parallel connection of inductor $L_{1}$ and capacitor $C_{1}$. $C_{p}$ and $C_{q}$ represent the coupling between the capacitive patch and the CP-DSIWR, and the parallel connection of $L_{2}$ and $C_{2}$ represents the external coupling of the CP-DSIWR.

**Fig 14:**
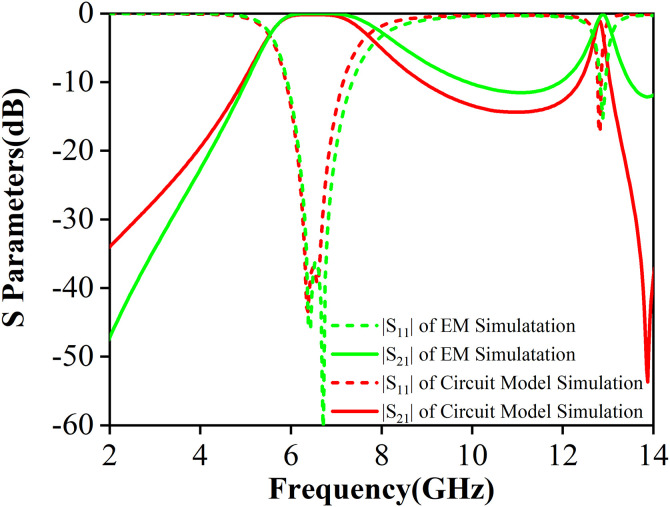
The S-parameters of equivalent-circuit model simulation and electromagnetic (EM) simulation.

As shown in Fig 14, for the dual-band mixed-mode bandpass filter, the electromagnetic (EM) simulation results for the S-parameters are as follows: the insertion losses of the two passbands is 0.16 dB and 1.12 dB respectively and the return losses of the simulations is better than 17 dB. The 3-dB bandwidths of the two passbands are 2.13 GHz and 0.17 GHz. The transmission zero is located at 13.87 GHz with −53.7 dB insertion loss. The S-parameters obtained from the equivalent circuit model match well with the electromagnetic simulation results, validating the accuracy of the equivalent circuit model.

The results confirm that the proposed dual-band mixed-mode bandpass filter achieves precise control over both center frequencies and bandwidths, demonstrating improved performance through effective resonant frequencies tuning. The final dimensions of the dual-band mixed-mode bandpass filter are identical to those of the mixed-mode bandpass filter, with the exception of the slot, characterized by the following parameters: $l_{x}=1$ mm, $l_{y}=6.9$ mm.

## 3 Fabrication and measurement

**Fig 15:**
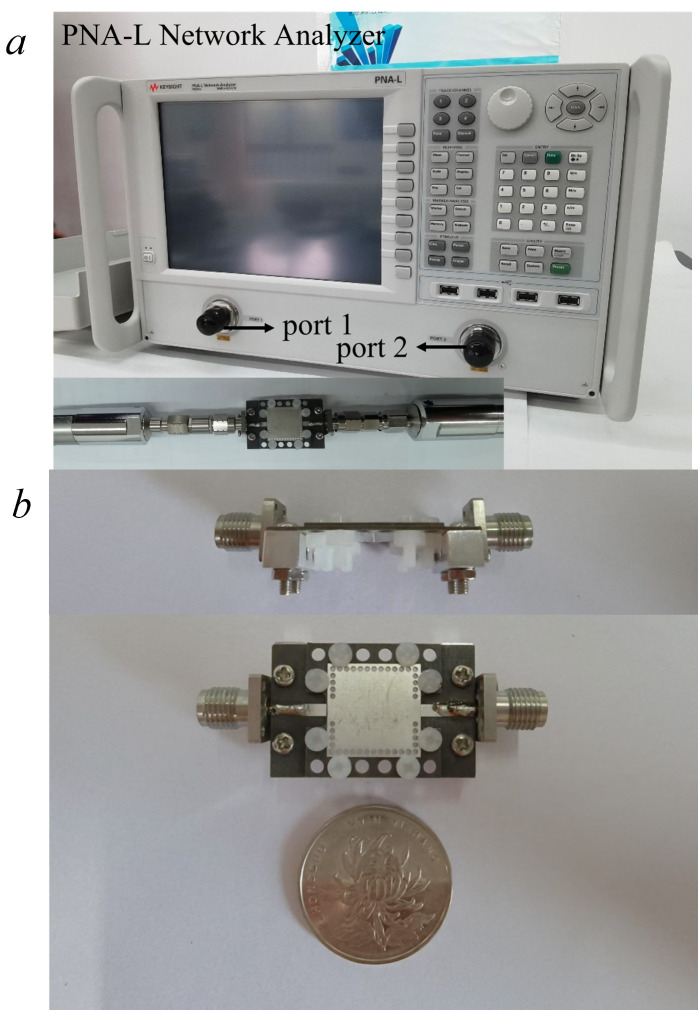
Fabrication and measurement. (a) The measurement setup; and (b) The filter prototype.

**Fig 16:**
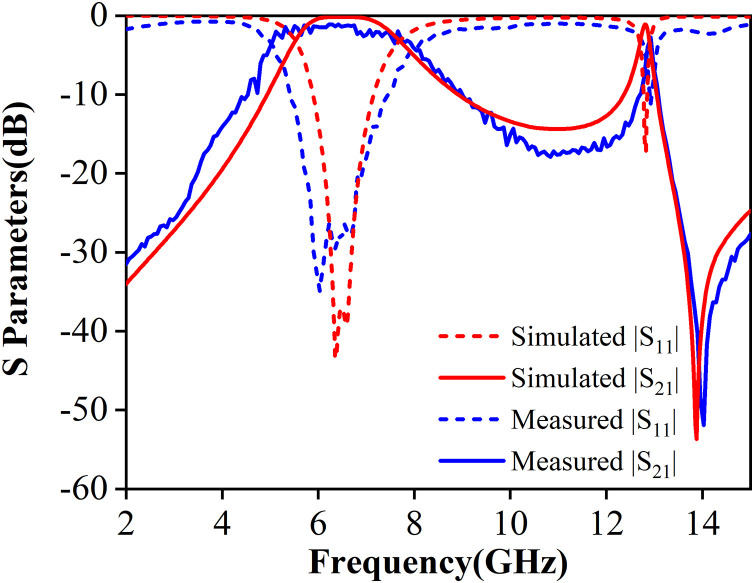
Simulation and measurement results of the proposed filter.

Fig 15(a) shows the photograph of the measurement setup, which includes a KEYSIGHT PNA-L Network Analyzer (Model: N5234A) with a frequency range of 10 MHz to 43.5 GHz, connected to the two 2.92 mm RF connectors of the filter prototype. Fig 15(b) shows the fabricated filter prototype, including its front view and top view. The dual-band mixed-mode bandpass filter is assembled by extending the PCB in the L-direction, with alignment holes and nylon screws to ensure precise layer stacking and stability during testing. The final fabricated filter has a compact size of 0.57 × 0.57$\lambda_{g}^{2}$. Fig 16 presents the simulation and measured results for the dual-band mixed-mode bandpass filter. The measured results indicate that the two passbands are centered at 6.2 GHz and 12.9 GHz with return losses exceeding 26 dB and 12 dB, respectively. The measured minimum in-band insertion losses of the two passbands are around 1.1 dB and 2.7dB. Machining accuracy and measurement tolerances can cause some slight deviations between the measured and simulated results.

## 4 Comparison and discussion

As shown in Table 2, compared to the dual-band filters reported in [12,16], the proposed dual-band mixed-mode bandpass filter can achieve bandwidth control. Compared to the dual-band filters in [8,10,12,17], the proposed filter coexists two types of modes, the patch mode and the cavity mode, with a higher richness of mode’s types and a larger number of modes to choose. The dimensions of the proposed filter are greatly reduced, occupying less than one-fourth of the size compared to those in [8,10,12,16,17]. Therefore, the size of the dual-band mixed-mode bandpass filter is significantly reduced compared to traditional SIW filters, demonstrating a substantial improvement in compactness. This is achieved by the use of a dual-layer structure and the loading of the capacitive patch without destroying the structure of the original resonator.

**Table 2:** Comparison with the reported state-of-the-art multi-band designs.

Ref.	MT	Tech	f_0_	NM	BC	Size	IL	RL
			(GHz)			($\lambda_{g}^{2}$)	(dB)	(dB)
[8]	1	SIW	13/14/15	3	Y	3.38 × 1.19	1.71/1.80/2.29	16.4/18.8/18
			11/12/13	3	Y	2.2 × 2.2	2.02/2.72/2.57	20/18.5/18.5
[10]	1	SIW	19.2	2	Y	1.15 × 1.15	0.5	22.1
			18.8/19.6	2	Y		0.68/0.61	24.8/17.4
			18.6/19.1/19.5	2	Y		0.98/0.67/1.4	22.7/20.1/19.2
[12]	1	SIW	12/16	2	N	1.92 × 1.92	1.07/0.95	>19
[16]	2	Hybrid	8.05/9.99	2	N	1.13 × 1.02	1.74/2.21	11.7/11.61
[17]	1	SIW	8.3/10.87	3	Y	1.06 × 1.06	1.9/1.71	>19.6
**This work**	2	Hybrid	6.47/12.8	3	Y	0.57 × 0.57	1.1/2.7	37/17.2

Ref: reference; MT: mode’s type; Tech: technology; f_0_: center frequency; NM: number of modes; BC: band control; $\lambda_{g}$: the guided wavelength at the center frequency of the fundamental cavity mode; IL: insertion loss; RL: return loss.

The proposed bandpass filter exhibits dual-band filtering characteristic. However, the stopband between the two passbands is not good, which will be improved in future work. Moreover, multiple modes will be exploited in multi-mode filters to enable the design of multi-band or wideband filters in the future.

## 5 Conclusion

Based on the SIW structure, this paper presents a dual-band mixed-mode bandpass filter utilizing a novel dual-layer substrate integrated waveguide resonator loaded with a capacitive patch, fed by a microstrip line. A detailed eigenmode analysis is conducted, and an experimental formula is derived. With metallic vias and slot embedded in the CP-DSIWR, a dual-band filter with tunable resonant frequencies is obtained, utilizing the TM_01_, TM_10_ and TE_101_ modes. This work breaks through the inherent idea of traditional cavity filters with only one type of resonant mode. Compared to traditional solutions, the proposed mixed-mode bandpass filter offers the advantage of mode controllability, providing a novel approach for designing dual-band or multi-band filter.

## Supporting information

S1 Data Simulation and measurement results. (CSV)
